# Methanethiol-dependent dimethylsulfide production in soil environments

**DOI:** 10.1038/ismej.2017.105

**Published:** 2017-08-01

**Authors:** Ornella Carrión, Jennifer Pratscher, Andrew R J Curson, Beth T Williams, Wayne G Rostant, J Colin Murrell, Jonathan D Todd

**Affiliations:** 1 School of Biological Sciences, University of East Anglia, Norwich, UK; 2 School of Environmental Sciences, University of East Anglia, Norwich, UK

## Abstract

Dimethylsulfide (DMS) is an environmentally important trace gas with roles in sulfur cycling, signalling to higher organisms and in atmospheric chemistry. DMS is believed to be predominantly produced in marine environments via microbial degradation of the osmolyte dimethylsulfoniopropionate (DMSP). However, significant amounts of DMS are also generated from terrestrial environments, for example, peat bogs can emit ~6 μmol DMS m^−2^ per day, likely via the methylation of methanethiol (MeSH). A methyltransferase enzyme termed ‘MddA’, which catalyses the methylation of MeSH, generating DMS, in a wide range of bacteria and some cyanobacteria, may mediate this process, as the *mddA* gene is abundant in terrestrial metagenomes. This is the first study investigating the functionality of MeSH-dependent DMS production (Mdd) in a wide range of aerobic environments. All soils and marine sediment samples tested produced DMS when incubated with MeSH. Cultivation-dependent and cultivation-independent methods were used to assess microbial community changes in response to MeSH addition in a grassland soil where 35.9% of the bacteria were predicted to contain *mddA*. Bacteria of the genus *Methylotenera* were enriched in the presence of MeSH. Furthermore, many novel Mdd^+^ bacterial strains were isolated. Despite the abundance of *mddA* in the grassland soil, the Mdd pathway may not be a significant source of DMS in this environment as MeSH addition was required to detect DMS at only very low conversion rates.

## Introduction

Dimethylsulfide (DMS) is a volatile compound with a significant impact on the environment owing to its roles in the global sulfur cycle, as an info-chemical and, potentially, in climate regulation. Microbes, particularly marine bacteria, drive the production of DMS, generating ~300 million tonnes *per annum* (Kettle and Andreae, 2000) through biotransformations of organosulfur molecules (Sievert *et al.*, 2007; Curson *et al.*, 2011). Most of the DMS produced is further degraded by bacteria or photochemical reactions in the oceans, but ~10% escapes into the atmosphere making it the most abundant biogenically derived form of sulfur transferred from the sea to the air (Kiene and Bates, 1990). DMS oxidation products initiate cloud formation over the oceans, on a scale that affects the sunlight reaching the Earth’s surface, which possibly affects climate through ‘global dimming’ (Sievert *et al.*, 2007; Vallina and Simó, 2007). However, the significance of DMS on climate regulation has been questioned (Quinn and Bates, 2011). When DMS, or its oxidation products, are delivered back to the Earth’s surface by precipitation, this represents a significant transfer of sulfur to land. DMS is also a chemoattractant for some seabirds, crustaceans and marine mammals that use it as a foraging cue (DeBose and Nevitt, 2008).

It is widely believed that DMS is mainly produced by microbial catabolism of the osmolyte dimethylsulfoniopropionate (DMSP) in marine environments via DMSP lyase enzymes (Curson *et al.*, 2011; Johnston *et al.*, 2016; Sun *et al.*, 2016). However, there are other routes for DMS production that are independent of DMSP and not limited to marine environments. These include the lysis of other dimethylsulfonium secondary metabolites or the enzymatic degradation of *S*-methyl-methionine, dimethylsulfoxide or methoxyaromatic compounds (Howard and Russell, 1996; Stets *et al.*, 2004; Spielmeyer *et al.*, 2011). These pathways are generally thought to be minor contributors to the global production of DMS. Conversely, many anaerobic marine, terrestrial and freshwater environments contain concentrations of DMS (1–44 nM; Lomans *et al.*, 1997) similar to, if not higher than DMS levels reported in the upper marine water column (Lana *et al.*, 2011), possibly resulting from the microbial methylation of methanethiol (MeSH; Kiene and Hines, 1995; Stets *et al.*, 2004), likely generated from methionine (Met) or H_2_S. These environments include saltmarsh sediments (Kiene and Visscher, 1987), freshwater lake sediments (Zinder and Brock, 1978; Lomans *et al.*, 1997, 2001), cyanobacterial mats (Zinder *et al.*, 1977) and peat bogs, some of which can emit ~6 μmol DMS m^−2^ per day (Kiene and Hines, 1995). The ability to generate MeSH from sulfide and/or Met under oxic conditions is very common in heterotrophic bacteria from diverse marine and terrestrial environments (Drotar *et al.*, 1987). Carrión *et al.*, 2015 showed that some marine and many terrestrial aerobic bacteria also methylate MeSH, generating DMS via a pathway termed MeSH-dependent DMS production (Mdd) and that these bacteria may contribute to the significant levels of DMS emitted by non-marine environments that were originally assigned only to anaerobic microbes.

Carrión *et al.*, 2015 identified the gene responsible for Mdd, termed ‘*mddA*’, in many bacteria. MddA is a membrane-bound *S*-adenosyl-Met-dependent methyltransferase that *S*-methylates MeSH, generating DMS. Functional MddA enzymes exist in taxonomically diverse and environmentally important bacteria, many of which had not been previously thought to make DMS. Examples comprise several genera of actinobacteria, including the pathogen *Mycobacterium tuberculosis*, Rhizobiales such as *Mesorhizobium* and *Bradyrhizobium* (many contain two distinct forms of MddA), nitrogen-fixing cyanobacteria and sediment-dwelling pseudomonads. The *mddA* gene is present in marine metagenomes such as the Global Ocean Sampling and the Monterey Bay microbial study, but, it is much more abundant in terrestrial environments and particularly in soils, where it is predicted to be present in up to 76% of bacteria (Carrión *et al.*, 2015). These soils include temperate grasslands, forest and farm soils, and the rhizosphere of rice. Given the prevalence of *mddA* in terrestrial metagenomes, rather than marine metagenomes, Carrión *et al.*, 2015 proposed that DMS produced from non-marine and, critically non-DMSP, sources may have a more significant role in global DMS production than previously thought. Here, we studied the functionality of the Mdd pathway in a wide range of environments and tested if the potential to release DMS from MeSH is greater in terrestrial, marine and/or freshwater environments. This represents the first step to evaluate the significance of this novel DMS-producing pathway in the environment. Focusing on a grassland soil with Mdd activity, we performed process measurements to study the activity of the Mdd pathway and the consumption of DMS produced by this process. We also combined cultivation-dependent and cultivation-independent microbial ecology techniques to identify the microbes involved in DMS production from MeSH in this grassland soil.

## Materials and methods

### Sample collection

Triplicate soil samples were collected from the oxic layer of two grassland soils (A and B), a forest soil and two agricultural soils (top 3 cm, Supplementary Table 1), after removing vegetation, using an ethanol-sterilised trowel. Triplicate lake, river and marine sediment samples (Supplementary Table 1) were collected from the oxic layer of sediment (top 3 cm) using an acrylic corer. Sand samples were collected from the oxic layer (top 3 cm) of two beaches (A and B, Supplementary Table 1) using an ethanol-sterilised trowel. 1 l of seawater was collected from each of two beaches (A and B, Supplementary Table 1) using a sterilised glass bottle. All samples were transferred immediately to the laboratory and kept at 4 °C until analysed. We focused on grassland soil A to perform the process measurements and the cultivation-dependent and molecular techniques described in this study.

### DMS production from MeSH by environmental samples

To study DMS production from MeSH by different environments, 1 g or 20 ml of sample was placed in a 125 ml serum vial containing distilled water supplemented with Y minimal medium 5% (Beringer, 1974), succinate (5 mM, Sigma-Aldrich, Dorset, UK) and MeSH added as sodium methanethiolate (20 μmol, Sigma-Aldrich) as described in Supplementary Methods. Additions of sodium methanethiolate will subsequently be referred to as additions of MeSH. All experiments were done in triplicate and vials were sealed prior to incubation at 22 °C for 24 h. MeSH and DMS headspace concentrations were measured by gas chromatography (GC) as described by Carrión *et al.*, 2015.

### Field measurements

Air-tight field chambers of 2 l volume were placed in the grassland soil studied and supplemented with MeSH (200 μmol) as described in Supplementary Methods. DMS and MeSH concentrations in the headspace were measured at *t*=0 and *t*=19 h by GC.

### Contribution of eukaryotes and prokaryotes to DMS production from MeSH

Microcosm experiments were supplemented with cycloheximide 200 μg ml^−1^ (Sigma-Aldrich) or with 100 μg ml^−1^ ampicillin, 50 μg ml^−1^ chloramphenicol, 5 μg ml^−1^ tetracycline and 400 μg ml^−1^ streptomycin (Sigma-Aldrich), incubated and assayed for Mdd by GC as described in Supplementary Methods. Vials with no antibiotics added were used as controls.

### Grassland soil enrichments with MeSH

**Figure 1:**
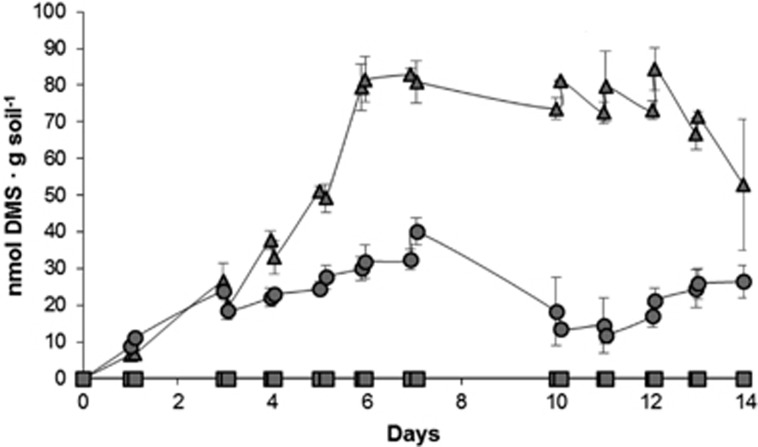
DMS in the grassland soil enrichments. DMS in the headspace of the grassland enrichments are expressed as nmol·g soil^−1^. MeSH (20 μmol) was added daily to the enrichments, with succinate plus MeSH or MeSH alone, for 14 days, as this gas was depleted after 24 h. Values represent the average of three biological replicates with their respective standard deviations. Triangles: enrichments with succinate plus MeSH; Circles: enrichments with MeSH; Squares: enrichments with succinate.

To study the effects of MeSH addition on the processes of DMS production and consumption, and on bacterial diversity, three different enrichments were each set up in triplicate. One set of enrichments was supplemented with succinate (5 mM). The second set of enrichments was supplemented with MeSH (20 μmol). The third set of microcosms was supplemented with succinate (5 mM) and MeSH (20 μmol). Vials were incubated at 22 °C for 14 days and supplemented daily with fresh MeSH to the corresponding samples as this gas disappeared after 24 h. MeSH and DMS in headspaces were monitored by GC as indicated on Figure 1 and in Supplementary Methods.

### Rates of MeSH consumption, DMS production and DMS consumption

Two sets of microcosms supplemented with succinate (5 mM) and MeSH (20 μmol) were set up in triplicate to estimate MeSH consumption and DMS production and consumption rates as detailed in Supplementary Methods. MeSH was added daily to both sets of microcosms for 14 days. At time 0, 7 and 14 days, one set of microcosms was supplemented with MeSH (20 μmol) to measure net MeSH consumption and DMS production rates by GC. At the same time points, DMS (0.5 μmol, Sigma-Aldrich) was added to the other set of microcosms to estimate net DMS consumption rates.

### MeSH and DMS consumption by *Methylotenera mobilis* JLW8^T^

*M. mobilis* JLW8^T^ cultures were incubated in vials containing MeSH (20 μmol) and DMS (272 nmol) for 24 h at 30 °C before measuring MeSH and DMS concentrations in the headspace by GC, as detailed in Supplementary Methods. Controls with medium and MeSH or DMS were set up and tested.

### Gas chromatography

GC was performed as described by Carrión *et al.*, 2015.

### Isolation and characterisation of strains

Samples from *t*=0 and those enriched with succinate plus MeSH for 14 days were plated onto Y minimal medium supplemented with succinate (5 mM) and MeSH (1 mM) as carbon sources. Colonies with different morphologies were purified and selected for further characterisation. Isolates were taxonomically identified by sequencing the 16S rRNA gene and characterised for their MeSH and DMS production as detailed in Supplementary Methods.

### Additional methods

For nucleic acids extraction from environmental samples, 16S rRNA gene amplicon sequencing, metagenomics and statistical analysis, see Supplementary Methods. 16S rRNA amplicon sequencing and metagenomics data is publicly available at NCBI single read archive (BioProject PRJNA390514).

## Results

### DMS production from MeSH in a grassland soil

To study the significance of the Mdd pathway in the environment, we initially analysed the ability of a local grassland soil with neutral pH (6.7) to produce DMS from MeSH. Soil samples incubated in the absence of MeSH produced no MeSH or DMS above our detection limits (0.2 nmol for DMS and 4 nmol for MeSH), likely indicating that MeSH is not abundant in this environment or that these gases are quickly consumed by the microbial population. However, samples incubated with MeSH (20 μmol) consumed it all in 24 h and produced 15.2±1.5 nmol DMS·g soil^−1^, which represents 0.1±0.01% of the added substrate. In control incubations with sterile soil supplemented with MeSH, MeSH disappeared but there was no DMS production. Chemical oxidation of MeSH to dimethyldisulfide (DMDS) was also studied, but there was no variation in DMDS in the soil samples (4.3±0.03 nmol, 0.02±<0.001% of the MeSH added) or sterile controls (4.2±0.2 nmol, 0.02%±0.001 of the MeSH added) over the incubation period. No other sulfurous compounds were detected in any of the samples. From these results, we conclude that the Mdd pathway is active in microorganisms in this grassland soil when MeSH is available.

### Field measurements

Having demonstrated that the Mdd pathway was active in the grassland soil samples in microcosm experiments, the sampling site was re-examined by placing two sets of 2 l air-tight field chambers over the soil. After 19 h, the results were congruent with those observed with serum vials, with the soil alone not making MeSH or DMS, but when it was supplemented with MeSH (200 μmol) the soil liberated 239.3±18.9 nmol of DMS (0.1±0.01% of MeSH added). Therefore, field measurements confirmed that microbes in the grassland soil had a functional Mdd pathway when MeSH is available.

### Contribution of eukaryotes and prokaryotes to DMS production from MeSH

Grassland soil samples were supplemented with antibiotics effective against either prokaryotes or eukaryotes and incubated for 24 h. The vials with prokaryotic inhibitors added showed a ~50% reduction in DMS (103.8±16.3 nmol DMS·g soil^−1^, 0.5±0.1% of the MeSH added) compared with the controls with no inhibitors added (231.4±13.3 nmol DMS·g soil^−1^, 1.2±0.1% of the MeSH added). In contrast, the eukaryotic inhibitor cycloheximide had no effect on DMS production (248.2±13.9 nmol DMS·g soil^−1^, 1.2±0.1% of the MeSH added), indicating that prokaryotes are probably the main contributors to DMS production from MeSH in this particular grassland soil.

### Grassland soil enrichments with MeSH

To identify the microbes responsible for DMS produced from MeSH in these grassland soil samples, we carried out microbial enrichment experiments. Soil samples were either supplemented with MeSH alone, succinate plus MeSH to study how DMS production is affected by carbon availability, or succinate alone to investigate changes in the microbial population owing to the added carbon source.

Enrichments with succinate alone showed no DMS or MeSH production above our detection limits (Figure 1), confirming the requirement for MeSH in this soil environment for the Mdd pathway to produce detectable amounts of DMS. Enrichments with MeSH alone showed progressive slight increases in DMS production over the first 7 days (to 32.7±2.8 nmol DMS·g soil^−1^). Subsequently, DMS production steadily declined (Figure 1). The enrichment with succinate plus MeSH showed a similar trend to the MeSH alone, with DMS production increasing rapidly over the first 6 days (to 79.3±6.4 nmol DMS·g soil^−1^), and then stabilising before DMS was more rapidly consumed after 11 days (Figure 1). Higher DMS production levels (~2.5-fold) were seen when MeSH was added in the presence of succinate. This is expected since when MeSH is added as sole carbon source, a higher proportion will be assimilated, especially when soil carbon reservoirs have been exhausted.

### Rates of MeSH consumption, DMS production and DMS consumption

To study in greater detail how the addition of MeSH affected the processes of DMS production and consumption, we measured both rates at 0, 7 and 14 days. For this, we used samples enriched with succinate plus MeSH as they showed the highest DMS production levels (see above). We also measured MeSH consumption rates at the same time points. Both MeSH consumption and DMS production net rates increased from *t*=0 (0.9±0.1 μmol MeSH·h^−1^·g soil^−1^; 0.4±0.1 nmol DMS·h^−1^·g soil^−1^) to 7 days (1.4±0.1 μmol MeSH·h^−1^·g soil^−1^; 4.0±1.4 nmol DMS·h^−1^·g soil^−1^) and reached maxima of 3.3±0.1 μmol MeSH·h^−1^·g soil^−1^ and 6.1±1.8 nmol DMS·h^−1^·g soil^−1^ at 14 days, confirming that extended exposure to MeSH enhances the activity of the Mdd pathway and may enrich for microbes producing DMS from MeSH. As DMS production rates increased over the course of the MeSH enrichment, it was not surprising to see that net DMS consumption rates also increased. The DMS consumption rate at *t*=0 was 0.2±0.01 nmol DMS·h^−1^·g soil^−1^, but after 7 days it was 3.9±1.0 nmol DMS·h^−1^·g soil^−1^, reaching a maximum of 10.1±1.9 nmol DMS·h^−1^·g soil^−1^ after 14 days. The fact that at 14 days DMS consumption was greater than DMS production indicates that there has probably been an enrichment of DMS-degrading microorganisms.

### Changes in the bacterial diversity in response to the enrichment of the grassland soil with MeSH

**Figure 2:**
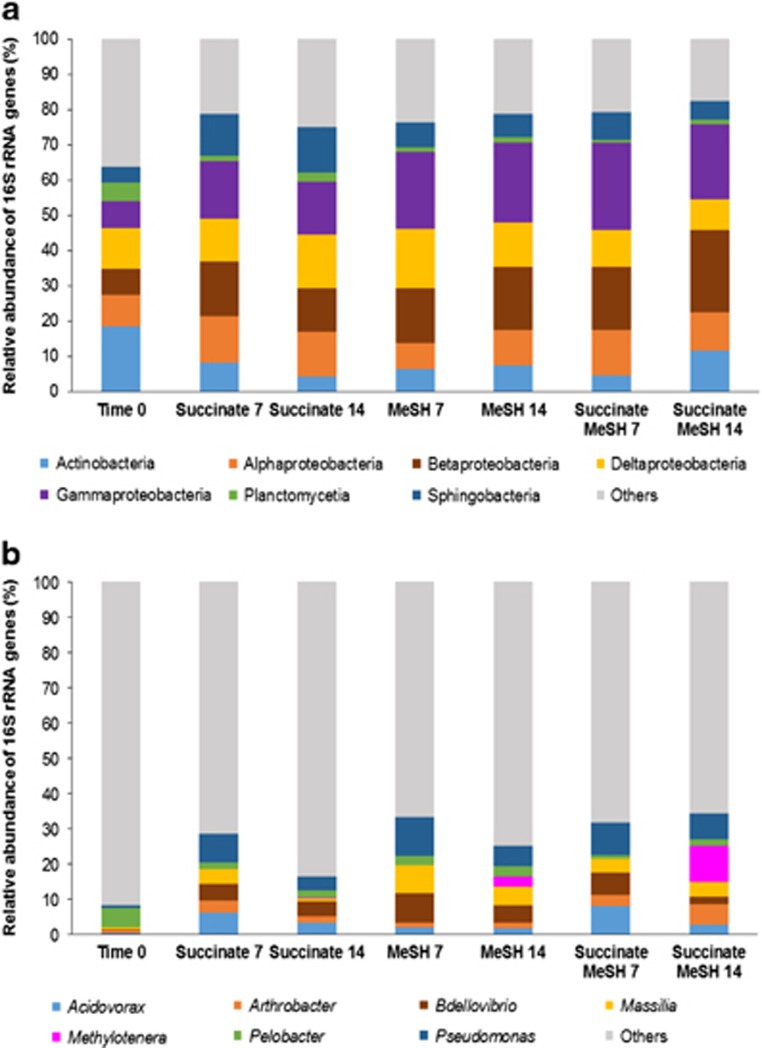
Taxonomic profiling of the 16S rRNA gene amplicon sequencing data from grassland soil enrichments. (**a**) Class level; (**b**) Genus level. Results shown are the average of two biological replicates. Only classes or genera that are ≥5% abundant in at least one of the conditions are represented. Time 0: grassland soil samples at time 0; Succinate 7: enrichments with succinate at 7 days, Succinate 14: enrichments with succinate at 14 days; MeSH 7: enrichments with MeSH at 7 days; MeSH 14: enrichments with MeSH at 14 days; Succinate MeSH 7: enrichments with succinate plus MeSH at 7 days; Succinate MeSH 14: enrichments with succinate plus MeSH at 14 days. Results for the individual replicates are shown in Supplementary Figure 1.

Having established that the MeSH incubations had probably enriched for microbes producing DMS from MeSH and also for microbes consuming MeSH and/or DMS, the resultant changes in microbial population were investigated. DNA was extracted from samples at *t*=0 and from enrichments with MeSH alone, succinate alone and succinate plus MeSH after 7 and 14 days of incubation. Extracted DNA was used as template in 16S rRNA gene amplicon sequencing (see Supplementary Methods). These sequencing data showed that the grassland soil natural population (*t*=0 samples) was dominated by bacteria from the classes Actinobacteria (18.5±0.9%) and Deltaproteobacteria (11.6±0.1%, Figure 2 and Supplementary Figure 1). The most abundant genus was *Pelobacter* (5.5±0.001%, Figure 2 and Supplementary Figure 1).

The addition of diluted Y medium with succinate as a carbon source (succinate-only enrichments) changed the profile of the natural microbial community with a significant increase in relative abundance of Gammaproteobacteria to 14.8±1.5% (Tukey honest significant difference (HSD), *P*-value=0.017), and marginally non-significant increases in Betaproteobacteria to 12.4±0.9% (Tukey HSD, *P*=0.060) and Sphingobacteria to 12.8±0.5% (Tukey HSD, *P*=0.055), after 14 days. Deltaproteobacteria were still one of the most predominant classes (15.4±1.7%), but Actinobacteria were substantially reduced to 4.2±0.04% (Tukey HSD, *P*=0.182; Figure 2 and Supplementary Figure 1). The genera *Acidovorax, Pseudomonas* and *Bdellovibrio* showed an increased relative abundance (up to 3.3±1.1%, *P*=0.004; 3.9±0.2%, *P*=0.032 and 4.2±0.3%, *P*=0.051, respectively, Tukey HSD) in the 14-day succinate enrichments compared with *t*=0 samples (Figure 2 and Supplementary Figure 1).

It was notable that MeSH addition to the grassland soil enrichments reduced the relative abundance of Sphingobacteria to 6.6±0.2% (Tukey HSD, *P*=0.293) in the 14-day MeSH enrichments and to 5.2±0.6% (Tukey HSD, *P*=0.100) in the succinate plus MeSH enrichments compared with the 14-day succinate samples. MeSH increased the relative abundance of Betaproteobacteria to 17.9±0.1% (Tukey HSD, *P*=0.228) in the 14-day MeSH enrichments and to 23.6±1.2% (Tukey HSD, *P*=0.018) in the succinate plus MeSH enrichments. The relative abundance of Gammaproteobacteria also increased to 22.8±3.8% in the 14-day MeSH enrichments (Tukey HSD, *P*=0.104) and to 23.6±2.1% (Tukey HSD, *P*=0.001) in the enrichments with succinate and MeSH compared with the 14-day enrichments with succinate (Figure 2 and Supplementary Figure 1). At the genus level, *Pseudomonas* (5.9±1.2% in the MeSH alone enrichments and 7.4±3.6% in the enrichments with succinate and MeSH) was still one of the most abundant genera in the bacterial community of the 14-day enrichments with MeSH. However, it was notable that the presence of MeSH significantly increased the relative abundance of *Methylotenera* and *Massilia* in the 14-day enrichments with MeSH (up to 2.8±0.9%, *P*=0.016 and 5.3±0.1%, *P*=0.002, respectively; Tukey HSD) and 14-day samples enriched with succinate plus MeSH (up to 10.3±1.5%, *P*=0.002 and 4.2±1.2%, *P*=0.006, respectively; Tukey HSD), compared with the 14-day enrichments with succinate. *Arthrobacter* also increased in relative abundance, although not significantly, in the 14-day enrichments with succinate plus MeSH (to 5.8±0.5%, Tukey HSD, *P*=0.181) compared with the 14-day succinate samples (Figure 2 and Supplementary Figure 1). The fact that members of these genera became more abundant after 14 days of incubation, when DMS consumption rates are maximal, suggests that they may have a role in DMS degradation.

Soil bacteria that are known to oxidise MeSH and DMS such as *Hyphomicrobium* and *Thiobacillus* were also present in the *t*=0 samples (0.3±0.04% and 0.8±0.1%, respectively), but their relative abundance did not increase either in the MeSH alone or the MeSH plus succinate enrichments.

DNA extracted from samples at *t*=0 and from enrichments with succinate plus MeSH after 7 and 14 days of incubation was also used to perform metagenomics (see Supplementary Methods). Phylogeny of the metagenomes was determined using MetaPhlAn. As MetaPhlAn uses a range of marker genes to assess the phylogeny of the reads, results slightly differed from those obtained from the 16S rRNA gene amplicon sequencing. According to the phylogenetic analysis of the metagenomes by MetaPhlAn, the bacterial population of the grassland soil at *t*=0 was dominated by members of the class Actinobacteria (51.4%), confirming the results of the 16S rRNA gene analysis. The most abundant bacteria in the *t*=0 community that MetaPhlAn could classify at a genus level belonged to *Agromyces* (13.7%), *Thiomonas* (8%), *Rhodopseudomonas* (6.7%) and *Pedobacter* (6.2%) genera (Supplementary Figure 2).

Consistent with the 16S rRNA gene amplicon sequencing data above, MetaPhlAn analysis indicated that the bacterial community of the 14-day enrichments with succinate and MeSH was dominated by Gammaproteobacteria (44.2%) and Betaproteobacteria (37.4%). The most abundant genus at this time point was *Pseudomonas* (41.6%, Supplementary Figure 2). Metagenomic analysis also showed that *Methylotenera* and *Massilia* increased in relative abundance at this time point (to 21.6% and 3%, respectively; Supplementary Figure 2), further supporting the results of the 16S rRNA gene amplicon sequencing analysis.

### MeSH and DMS consumption by *M. mobilis* JLW8^T^

*M. mobilis* JLW8^T^, which is >97% identical at the 16S rRNA gene level to the *Methylotenera* in our MeSH enrichments, was tested for its ability to degrade MeSH and DMS. Cultures of *M. mobilis* JLW8^T^ were supplemented with MeSH or DMS and the amount of these gases in the headspace was quantified by GC at *t*=0 and 24 h. After 24 h of incubation, *M. mobilis* consumed 543.6±11.2 nmol MeSH (93.4±0.3%, Supplementary Table 2). *M. mobilis* also removed 1.1±0.02 nmol DMS (96.4±0.5%) in the first 24 h (Supplementary Table 2). These results show that *Methylotenera* can catabolise MeSH and DMS. However, when tested, *M. mobilis* could not use DMS or MeSH as sole carbon source (data not shown).

### Isolation and characterisation of strains

To complement cultivation-independent work, we isolated bacteria from grassland soil samples at *t*=0 and from enrichments supplemented with succinate plus MeSH after 14 days of incubation. These enrichments were chosen because the presence of both succinate and MeSH led to the greatest levels of DMS production and also the samples at 14 days had the highest rates of Mdd and consumption.

Strains isolated from the *t*=0 samples were Gammaproteobacteria (*Pseudomonas* spp.), Actinobacteria (*Streptomyces* spp.) and Bacilli (*Bacillus* spp., Supplementary Figure 3 and Supplementary Table 3). This is consistent with 16S rRNA gene amplicon sequencing analysis, which showed that Actinobacteria and Gammaproteobacteria were abundant in the *t*=0 grassland soil bacterial population (18.5±0.9% and 7.5±1.0%, respectively, Figure 2). Phylogeny of the isolates was also congruent with the sequencing data at the genus level, as these microorganisms were present in the *t*=0 samples according to the 16S rRNA gene analysis (*Pseudomonas* spp., 0.8±0.2% *Streptomyces* spp., 0.6±0.1% and *Bacillus* spp., 0.5±0.1%).

Isolates from enrichments with succinate plus MeSH at *t*=14 days were mainly Alphaproteobacteria (*Gemmobacter* spp., *Phyllobacterium* spp., *Rhizobium* spp., *Sinorhizobium* spp. and *Ensifer* spp.), but also Gammaproteobacteria (*Pseudomonas* spp. and *Acinetobacter* spp.), with *Pseudomonas* being the most abundant genus isolated (Supplementary Figure 3 and Supplementary Table 3). Isolation of Alpha- and Gammaproteobacterial strains was not surprising given the abundance of these classes in the succinate plus MeSH enrichments at 14 days according to the 16S rRNA gene amplicon sequencing data (10.6±0.1% and 21.1±2.1%, respectively, Figure 2). At the genus level, these microorganisms were also found in the 16S rRNA gene amplicon sequencing data from the succinate and MeSH enrichments at *t*=14 days with the following relative abundance: *Gemmobacter* spp., 0.002±0.0002% *Phyllobacterium* spp., 0.02±0.001% *Rhizobium* spp., 0.3±0.1% *Sinorhizobium* spp., 0.02±0.002% *Ensifer* spp., 0.1±0.003; *Pseudomonas* spp., 7.4±3.6% and *Acinetobacter* spp., 0.8±0.2%.

Isolates were tested for DMS production in Y minimal medium alone or Y medium supplemented with Met (a precursor of MeSH) or MeSH. Only ~5% of the isolates from *t*=0 samples were able to produce DMS from Y medium alone. However, ~58% of the isolates generated DMS when provided with Met or MeSH (Supplementary Table 3). These results highlight that diverse bacteria of the natural grassland soil bacterial population possess the ability to activate the Mdd pathway when MeSH or MeSH precursors such as Met are available.

When isolates from the succinate plus MeSH enrichments (14 days) were analysed, ~42% were able to produce DMS from Y medium alone. This percentage increased to ~96% and 100% of the isolates generating DMS when Y medium was supplemented with Met or MeSH, respectively (Supplementary Table 3). These results indicate that the enrichments with succinate and MeSH efficiently selected for bacteria that use the Mdd pathway. None of the isolates could use MeSH or DMS as sole carbon sources (data not shown).

### Abundance of genes encoding DMS-producing and DMS-degrading enzymes in the metagenomes of the grassland soil

DNA extracted from grassland soil samples at *t*=0 and from enrichments with succinate plus MeSH after 7 and 14 days of incubation was subjected to metagenomic analysis (see Supplementary Methods). Data obtained were analysed to study the changes in the relative abundance of genes encoding the MddA protein, as well as the enzymes involved in DMS degradation DdhA (DMS dehydrogenase, McDevitt *et al.*, 2002), DmoA (DMS monooxygenase, Boden *et al.*, 2011) and Tmm (Trimethylamine monooxygenase that oxidises DMS to DMSO, Lidbury *et al.*, 2016) to relate them to the DMS production and consumption rates observed over the course of grassland soil enrichments with succinate plus MeSH. The *megL* gene, which encodes a Met gamma lyase that cleaves Met to MeSH (Tanaka *et al.*, 1976), was also screened to determine the potential of the bacterial population to generate MeSH from Met (see Supplementary Methods for details and criteria to identify genes).

Metagenomic data showed that *megL* was very abundant in the grassland soil bacteria at *t*=0 (78% of bacteria, Supplementary Table 4), indicating that the ability to produce MeSH from Met resides in the natural microbial community of this environment. Furthermore, 35.9% of bacteria contained genes encoding MddA and 18.2% contained genes involved in DMS degradation (*ddhA, dmoA* and/or *tmm*, Supplementary Table 4). The relatively high occurrence of these genes in the natural soil bacterial population together with the DMS production and consumption rates observed above, suggest that the processes of Mdd and DMS degradation are likely important under certain conditions that this soil encounters.

The analysis of the metagenomes from the succinate plus MeSH enrichments showed a decrease in the percentage of bacteria containing *mddA* at 7 days (19.5%) and, although increased up to 25.3% at 14 days, was still lower than *t*=0 (Supplementary Table 4). Genes involved in DMS degradation were present in 12.6% of bacteria at 7 days and decreased to 9% at 14 days (Supplementary Table 4). Conversely, DMS production and consumption rates increased over the course of the enrichments, being 15- and 50-fold higher at 14 days than at *t*=0, respectively. The discrepancies observed between the relative abundance of the target genes analysed and the process rates indicate that there probably are other bacterial enzymes involved in DMS production and consumption in this grassland soil.

### Diversity of *mddA* in grassland soil metagenomes

Metagenomes from samples at *t*=0 and samples from enrichments with succinate and MeSH at 7 and 14 days were analysed for changes in the diversity of bacterial *mddA* due to the addition of MeSH.

**Figure 3:**
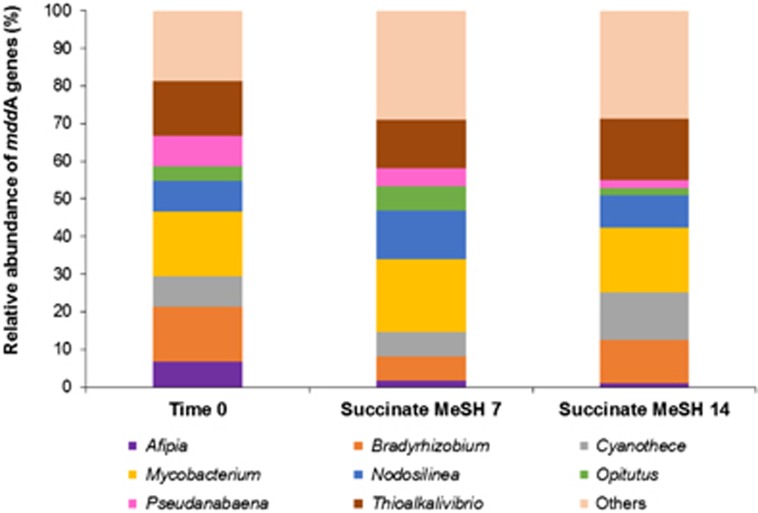
Diversity and relative abundance of *mddA* genes in the grassland soil unassembled metagenomes. Time 0: grassland soil samples at time 0; Succinate MeSH 7: enrichment with succinate plus MeSH at 7 days; Succinate MesH 14: enrichment with succinate plus MeSH at 14 days.

In the unassembled metagenomes, the most abundant *mddA* genes in *t*=0 samples were closely related to those from *Bradyrhizobium*, *Mycobacterium* and *Thioalkalivibrio*, which accounted for 14.7%, 17.3% and 14.7% of the *mddA*-containing bacteria, respectively (Figure 3). After 7 days of enrichment with succinate and MeSH, the relative abundance of the *Bradyrhizobium mddA* homologues decreased to 6.5%, but the *Mycobacterium* (19.4%) and *Thioalkalivibrio* (12.9%) forms were still the most abundant *mddA*-like genes together with *Nodosilinea mddA* homologues (12.9%, Figure 3). *Mycobacterium* and *Thioalkalivibrio* forms were also the most abundant *mddA* genes in the 14-day samples (17.3% and 16.3%, respectively), followed by *Cyanothece* (12.5%) and *Bradyrhizobium* (11.5%) *mddA*-like genes (Figure 3). Therefore, these data suggest that the diversity of the *mddA* gene did not change significantly throughout the enrichments.

**Figure 4:**
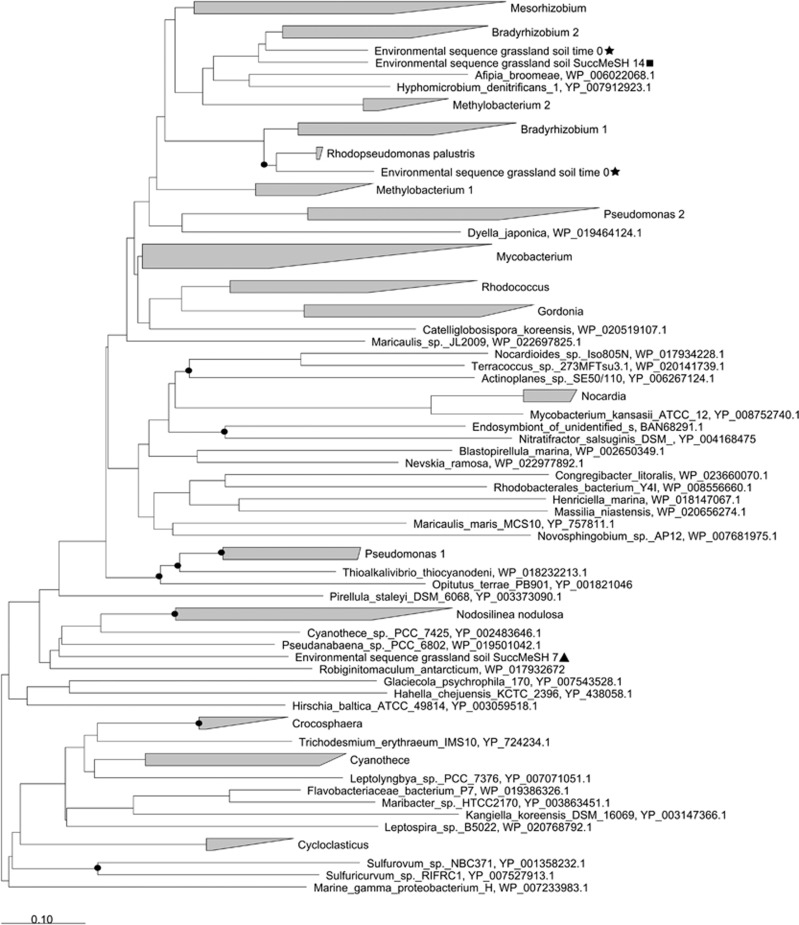
Neighbour-joining phylogenetic tree of *mddA* including the environmental sequences from the assembled metagenomes of the grassland soil. Stars: sequences obtained from the metagenome of the grassland soil at time 0; Triangles: sequences obtained from the metagenome of the succinate plus MeSH enrichment after 7 days; Squares: sequences obtained from the metagenome of the succinate plus MeSH enrichment after 14 days. The accession numbers of the proteins encoded by the nucleotide sequences used in this tree are shown. Sequences with 57% identity to each other that are in bacteria of the same genus are shown in triangles; the size of the triangle reflects the number of sequences. Bar, 0.10 substitutions per nucleotide position. Bootstrap values ⩾70% (based on 1000 replicates) are represented with dots at branch points.

Analysis of the assembled metagenomes also showed that the *mddA* genes from *t*=0 and *t*=14 days samples were closely related to *Bradyrhizobium* forms, whereas sequences from *t*=7 days samples were more similar to *Nodosilinea mddA-*like genes (Figure 4).

### Functionality of the Mdd pathway in other environments

To determine whether the Mdd pathway was functional in a wider range of environments, we analysed different terrestrial, freshwater and marine samples (Table 1). After 24 h of incubation, none of the samples incubated in the absence of MeSH produced DMS at detectable levels. However, all the soils and marine sediments tested produced DMS when supplemented with MeSH (20 μmol, Table 1). It is notable that the highest Mdd activities were observed with the samples from the terrestrial environments (Table 1). No Mdd activity was detected in any of the freshwater sediments, sand or seawater samples, suggesting that the Mdd pathway is less prevalent in these environments or that it may have been masked by DMS consumption processes. Seawater samples were also incubated in the presence of DMSP (20 μmol) and these released 5.5±0.6 nmol DMS·ml^−1^ (0.03±0.003% of DMSP added, Seawater A) and 25.8±4.7 nmol DMS·ml^−1^ (0.13±0.023% of DMSP added, Seawater B), respectively, indicating that DMSP is probably a more effective precursor for DMS than MeSH in these environments.

## Discussion

The functionality of the Mdd pathway was studied for the first time in a wide range of terrestrial, freshwater and marine environments. MeSH addition led to the production of DMS in all tested soils and marine sediments. However, DMS was only detected when external MeSH was added. This may be due to the generated gases being subsequently rapidly degraded by microorganisms, but it could also be a consequence of only analysing the oxic layer in the microcosms experiments. Therefore, MeSH generated from the methylation of H_2_S in the anaerobic layers of the soil and sediments (Drotar *et al.*, 1987; Lomans *et al.*, 1997) would be absent. The vegetation above the soil was removed before sampling, thus, the soil microcosms would also lack much of the MeSH emitted by plants (Schmidt *et al.*, 1985; Rennenberg, 1991; Boerjan *et al.*, 1994; Saini *et al.*, 1995). Nevertheless, our findings indicate that the Mdd pathway is not likely a significant source of DMS in the tested environments. It is possible that in other environments with high sulfur content or protein degradation levels, such as decaying vegetation, sewage, algal mats or manure, where MeSH concentrations may be significantly higher (Zinder and Brock, 1978; Drotar *et al.*, 1987), the Mdd pathway would generate high concentrations of DMS without MeSH additions. Thus, it would be interesting to study these environments in the future to confirm this hypothesis.

No freshwater sediments, beach sands or seawaters tested produced detectable amounts of DMS after 24 h of incubation, even with MeSH added, suggesting that the Mdd pathway is not present or functional in these environments. However, we cannot rule out the possibility that the Mdd pathway could be active, but the DMS consumption rates are greater. This also would be congruent with observations in many marine and freshwater environments where DMS formed is rapidly degraded by microorganisms (Kiene and Visscher, 1987; Kiene and Bates, 1990; Lomans *et al.*, 1997; Stets *et al.*, 2004; Lyimo *et al.*, 2009). Therefore, a detailed monitoring of Mdd and consumption processes together with molecular microbial ecology studies are needed to assess more precisely the significance of the Mdd pathway in freshwater and marine environments.

Another pointer for the Mdd pathway not being a significant source for DMS in the tested environments was our finding that only a small percentage (~0.1%) of the MeSH added to our microcosm experiments was captured as DMS despite all the MeSH disappearing within 24 h. Some of the MeSH added may have been enzymatically degraded to H_2_S and formaldehyde by methanethiol oxidase-containing bacteria (Suylen *et al.,* 1987; Gould and Kanagawa, 1992; Kim *et al.,* 2000; Lee *et al.,* 2002; Boden *et al.*, 2011). In addition, MeSH can be abiotically removed as shown by controls with sterile soil. It is known that MeSH can be chemically oxidised to DMDS (Bentley and Chasteen, 2004; Kiene, 1996). DMDS was detected in our experimental microcosms and sterile soil controls, but did not accumulate over the incubation period. Thus, abiotic oxidation to DMDS does not likely account for high proportions of MeSH disappearance in our experiments and no other sulfurous compounds were detected in any of the samples. Therefore, we propose that a significant proportion of MeSH removal in the sterile controls and experimental samples is likely owing to its reaction with particles and dissolved organic matter, as previously described by other authors (Zinder and Brock, 1978; Kiene and Hines, 1995; Kiene, 1996).

The biotic and abiotic degradation of MeSH greatly affects the amount of this compound available for microorganisms that use the Mdd pathway to produce DMS. A further contributing factor to the low levels of DMS achieved in our microcosm experiments is the biotic and abiotic degradation of DMS. The rates of DMS consumption increased over the course of the succinate plus MeSH enrichments to levels above those for Mdd after 14 days. DMS can be photochemically (Hatton, 2002) and enzymatically (McDevitt *et al.*, 2002; Lidbury *et al.*, 2016) oxidised to dimethylsulfoxide. DMS can also be metabolised by bacteria containing DMS monooxygenase (DmoA) to produce MeSH (Boden *et al.*, 2011). Indeed, the genetic potential to carry out biological DMS oxidation (via *dmoA, ddhA* and *tmm*) was relatively abundant in the natural grassland soil bacterial population. However, the relative abundance of *dmoA, ddhA* and *tmm* did not increase over the succinate plus MeSH enrichments despite a 50-fold increase in DMS consumption rate, perhaps suggesting that there are as yet unidentified microbial genes for DMS degradation. In any case, it is clear that the DMS production levels reported here are an underestimation of the activity of the Mdd pathway in the grassland soil studied.

Although we have shown that the ability to generate DMS from MeSH is inherent in all of the different soils and marine sediments tested, it is still very difficult to estimate the exact contribution of the Mdd pathway to global DMS emissions, since there are very few *in situ* measurements of MeSH and none of them from soils (Ulshöfer *et al.*, 1996; Lomans *et al.*, 1997; Kettle *et al.*, 2001; Xu *et al.*, 2001), and estimates of DMS produced from terrestrial environments are also scarce (Watts, 2000).

The addition of MeSH to the grassland soil samples not only increased DMS production and consumption rates, but also changed the diversity of the bacterial population. According to cultivation-independent methods, relative abundance of *Methylotenera* significantly increased in 14-day enrichments supplemented with MeSH, when DMS consumption rates were maximal. This indicates that *Methylotenera* likely has a role in DMS degradation, as suggested by Eyice *et al.*, 2015. The potential of members of the genus *Methylotenera* to remove DMS was confirmed when test cultures of *M. mobilis* JLW8^T^ consumed both DMS and MeSH. To our knowledge, this represents the first proof that members of the *Methylophilaceae* family, which includes obligate and facultative methylotrophs, are able to degrade these volatile compounds. Increases in the relative abundance of *Arthrobacter* genera in the 14-day succinate plus MeSH enrichments, suggests that they may also be involved in DMS consumption. Indeed, some *Arthrobacter* strains are able to metabolise DMS to generate MeSH (Borodina *et al.*, 2000). It is also known that other soil inhabitants such as *Hyphomicrobium* or *Thiobacillus* can oxidise MeSH and DMS (Cho *et al.*, 1991; Zhang *et al.*, 1991; Visscher and Taylor, 1993; Boden *et al.*, 2011). However, these microorganisms were present at very low relative abundance levels in the *t*=0 samples, and did not increase over the course of the MeSH enrichments, suggesting that they may not have a significant role in the removal of MeSH and DMS in our microcosms experiments. Finally, 16S rRNA gene amplicon sequencing data and metagenomics analysis also showed that the presence of MeSH increased the relative abundance of *Massilia*, some of which contain *mddA*-like genes (Carrión *et al.*, 2015). Further studies are required to confirm the role of *Massilia* in DMS production.

Cultivation-dependent methods showed that the addition of MeSH to grassland soil samples selected for microorganisms containing the Mdd pathway. Most of the isolates that generated DMS from MeSH belonged to the genus *Pseudomonas*, which is not surprising, given that many contain *mddA* (Carrión *et al.*, 2015). However, we also isolated strains of *Acinetobacter, Gemmobacter, Phyllobacterium, Rhizobium, Ensifer* and *Sinorhizobium* that converted MeSH into DMS. These bacteria were not previously suspected to have *mddA* or produce DMS from MeSH, since close relatives with sequenced genomes lack this gene. Thus, the Mdd pathway is more widespread among different bacterial taxa than previously thought. It is probable that other Mdd enzymes drive DMS production from MeSH in these bacteria. However, detailed molecular characterisation of these microorganisms is required to identify their Mdd enzymes and ultimately prove this. The presence of other Mdd enzymes in the bacterial population of the grassland soil could explain the observed discrepancies between metagenomics analysis and process rates, where the relative abundance of *mddA* did not increase throughout the enrichments despite the increase in DMS production rates. Such differences could also be due to the expression levels of the genes encoding the diverse Mdd proteins and/or their respective enzyme activity. Future studies will need to consider these factors to ascertain at a molecular level how and why Mdd levels change.

In conclusion, we show that the Mdd pathway is present and active when MeSH is available in a wide range of terrestrial and marine environments, especially in soils. However, as only ~0.1% of MeSH added is transformed into DMS via this pathway, it is not likely a significant process in the tested environments under natural conditions. More microbiological studies and process-based measurements are required on environments where MeSH is likely abundant to make an accurate estimation of the contribution of the Mdd pathway to the global DMS cycle.

## Figures and Tables

**Table 1:** DMS production by samples from different terrestrial and aquatic environments after 24 h of incubation with MeSH (20 μmol)

*Environment*	*nmol DMS per g or per ml of sample*	*% of DMS produced from MeSH*
Grassland soil A	15.2±1.5	0.08±0.007
Grassland soil B	19.6±0.6	0.10±0.003
Forest soil	14.9±0.7	0.07±0.003
Maize field soil	9.5±0.7	0.05±0.004
Barley field soil	5.5±0.7	0.03±0.003
Marine sediment A	6.4±0.8	0.03±0.004
Marine sediment B	5.0±0.1	0.03±0.001
River sediment	ND	ND
Lake sediment	ND	ND
Beach sand A	ND	ND
Beach sand B	ND	ND
Seawater A	ND	ND
Seawater B	ND	ND

DMS production is expressed as nmol DMS per g sample or nmol DMS per ml sample in case of seawater samples. Values shown are the average of three biological replicates with respective standard deviations ND, not detected.
