# Supplementary material for: Methanethiol-dependent dimethylsulfide production in soil environments
Source: ISME J. 2017 Aug 1;11(10):2379–90. doi: 10.1038/ismej.2017.105 (PMC5607357; doi:10.1038/ismej.2017.105)
Supplement: Supplementary Information [file ismej2017105x1.docx]

**Title: Methanethiol-dependent dimethylsulfide production in soil environments**

Ornella Carrión^1^, Jennifer Pratscher^2^, Andrew R. J. Curson^1^, Beth T. Williams^1^, Wayne G. Rostant^1^, J. Colin Murrell^2^, Jonathan D. Todd^1*^

^1^School of Biological Sciences, University of East Anglia, Norwich NR4 7TJ, UK

^2^School of Environmental Sciences, University of East Anglia, Norwich NR4 7TJ, UK

^*^Correspondence: [jonathan.todd@uea.ac.uk](mailto:jonathan.todd@uea.ac.uk)

**Supplementary Information**

**File content**

This file contains additional methods and supplementary figures, tables and references.

**Supplementary Methods**

DMS production from different environments

To study the DMS produced from soils, sediments and sands, 1 g of sample (in triplicate) was placed in a 125 ml serum vial containing 20 ml of distilled water supplemented with Y minimal medium 5% (Beringer, 1974), succinate (5 mM, Sigma-Aldrich, Dorset, UK) and MeSH added as sodium methanethiolate (20 µmol, Sigma-Aldrich). Additions of sodium methanethiolate will subsequently be referred to as additions of MeSH. To study DMS production from MeSH in seawater samples, microcosms experiments were set up in 125 ml serum vials containing 20 ml of seawater, Y medium 5%, succinate (5 mM) and MeSH (20 µmol). DMS production from DMSP in seawater samples was studied by supplementing vials containing 20 ml seawater, Y medium 5% and succinate (5 mM) with DMSP (20 µmol). Succinate was added as an additional carbon source to promote growth of bacteria in samples and avoid carbon depletion during incubation. Vials without MeSH or DMSP added were used as controls to determine if samples produced MeSH or DMS under natural conditions. Samples from the same environments were autoclaved twice (stored at -80 ºC until use) and used as controls to show that variation of MeSH or DMS concentrations in the headspace was due to microbial activity. All experiments were done in triplicate and vials were sealed prior to incubation at 22 ºC for 24 h. MeSH and DMS headspace concentrations were measured by gas chromatography (GC) as described by Carrión *et al*., 2015.

Field measurements

Air-tight field chambers of 2 L volume were placed in an area of 2.0 x 1.5 m (4 cm deep) of the grassland soil studied. MeSH (200 µmol) was added to three chambers and another three chambers with no MeSH addition were used as controls to determine if the soil produced MeSH or DMS under natural conditions. DMS and MeSH concentrations in the headspace of chambers were measured at 0 h and at 19 h by GC.

Contribution of eukaryotes and prokaryotes to DMS production from MeSH

To study the contribution of eukaryotes and prokaryotes to DMS production from MeSH in the grassland soil, vials (as above) were supplemented with cycloheximide 200 µg·ml^-1^ (Sigma-Aldrich) or with 100 µg·ml^-1^ ampicillin, 50 µg·ml^-1^ chloramphenicol, 5 µg·ml^-1^ tetracycline and 400 µg·ml^-1^ streptomycin (Sigma-Aldrich), respectively. Vials with no added antibiotics were used as controls. Vials from all the different conditions were set up in triplicate and incubated sealed at 22 ºC for 24 h before measuring DMS production by GC.

Grassland soil enrichments with MeSH

To study the effects of MeSH addition on the processes of DMS production and consumption, and on bacterial diversity in the grassland soil samples, three different enrichment experiments were each set up in triplicate. All contained 1 g of grassland soil, 20 ml of distilled water and Y medium (5%). One set of enrichments was supplemented with succinate (5 mM) to determine how the presence of an additional carbon source affected the diversity of the bacterial community. The second set of enrichments was supplemented with MeSH (20 µmol) to determine which changes in the bacterial community were dependent on the presence of MeSH and to study the functionality of the Mdd pathway. The third set of enrichments was supplemented with succinate (5 mM) plus MeSH (20 µmol) to study how the Mdd pathway was affected by carbon availability. Sterile controls were used to follow abiotic effects. All vials were sealed and incubated at 22 ºC for 14 days. MeSH and DMS concentrations in headspaces were monitored by GC as indicated on Figure 1. Vials were briefly opened every day to avoid oxygen depletion and to add fresh MeSH (20 µmol) to the corresponding samples, as this gas disappeared after 24 h.

Rates of MeSH consumption, DMS production and DMS consumption

Two sets of microcosms supplemented with succinate (5 mM) and MeSH (20 µmol) were set up in triplicate to estimate MeSH consumption and DMS production and consumption rates as those containing succinate plus MeSH showed the greatest Mdd activity. MeSH was added daily to both sets of microcosms for 14 days to study how the Mdd pathway affected DMS production and consumption processes. At time 0, 7 and 14 days, one set of microcosms was supplemented with MeSH (20 µmol) to measure net MeSH consumption and DMS production rates by GC. At the same time points, DMS (0.5 µmol, Sigma-Aldrich) was added to the other set of microcosms in order to estimate net DMS consumption rates. DMS disappearance was monitored by GC. Vials with sterile soil were used as controls. Net rates of DMS production and consumption are expressed as nmol·h^-1^·g soil^-1^. Net rates of MeSH consumption are expressed as µmol·h^-1^·g soil^-1^.

MeSH and DMS consumption by *Methylotenera* *mobilis* JLW8^T^

*Methylotenera mobilis* JLW8^T^ was obtained from M. G. Kalyuzhnaya (Kalyuzhnaya *et al.*, 2006). Starter cultures of *M. mobilis* JLW8^T^ were set up in variant-Hypho (vH) medium (Delaney *et al.*, 2013) supplemented with methylamine (10 mM, Sigma-Aldrich) and grown for 72 h at 30 ºC. Starter cultures were used to inoculate 125 ml serum vials containing 20 ml of fresh vH medium. Vials were then supplemented with MeSH (20 µmol) or DMS (0.3 µmol). Controls with medium and MeSH or DMS were set up and tested. Vials were incubated sealed at 30 ºC for 24 h before measuring MeSH and DMS concentrations in the headspace by GC.

For sole carbon source growth tests, *M. mobilis* JLW8^T^ was grown for 72 h at 30 ºC in vH medium with methylamine (10 mM). Cultures were spun down and pellets were washed three times with medium containing no carbon source. Pellets were then resuspended in medium with no carbon and adjusted to an OD_600_ of 0.6. This suspension was used to inoculate fresh vH medium supplemented with no carbon source or methylamine, MeSH, DMS, each at a concentration of 2 mM. Cultures were incubated for seven days before estimating cell density at OD_600_ with a UV-1800 spectrophotometer (Shimadzu, Milton Keynes, UK).

Isolation and characterisation of strains

Samples from time 0 (t=0) and samples enriched with succinate plus MeSH for 14 days were serially diluted and plated onto Y minimal medium supplemented with succinate (5 mM) and MeSH (1 mM) as carbon sources. Plates were incubated at 28 ºC and after 24-72 h, single colonies were obtained. Colonies with different morphologies were purified and selected for further characterisation.

For identification, the 16S rRNA gene from each isolate was amplified using the primer set 27F/1492R (Delong, 1992; Lane *et al.*, 1985). Purified PCR products were sequenced by Eurofins Genomics (Munich, Germany) and isolates were taxonomically identified using BLASTn ([http://blast.ncbi.nlm.nih.gov](http://blast.ncbi.nlm.nih.gov/Blast.cgi)).

To measure DMS and MeSH produced by isolates, cells were grown overnight in Y medium with succinate (5 mM) as a carbon source. Cultures were then adjusted to an OD_600_ of 0.3 and diluted 10-fold into 300 µl of Y medium supplemented with Met (0.5 mM), MeSH (0.3 µmol) or no substrate. Vials were incubated overnight at 30 ºC before measuring the concentration of MeSH and DMS in the headspace by GC. Cellular protein content was estimated by Bradford assays (BioRad, Hemel Hempstead, UK). Rates of MeSH and DMS production are expressed as nmol·min^-1^·mg protein^-1^.

To determine if isolates could use MeSH and/or DMS as sole carbon sources, they were grown overnight at 30 ºC in Y medium with succinate (5 mM) as carbon source. Cultures were pelleted and washed three times with Y medium without any carbon source and finally adjusted to an OD_600_ of 0.6. Cultures were then inoculated into fresh Y medium containing no carbon source, succinate, MeSH or DMS, each at a concentration of 2 mM. Cultures were incubated at 28 ºC for 96 h and growth was estimated by measuring cell density at OD_600_. All tests were performed in triplicate and repeated at least twice.

DNA and RNA extraction from environmental samples

DNA and RNA were extracted from the grassland soil samples at t=0 and from samples enriched with succinate (5 mM) alone, MeSH (20 µmol) alone and succinate plus MeSH at 7 and 14 days. To extract nucleic acids, 0.5 g of sample was added to a 2 ml screw-cap tube containing 0.1 mm silica beads (MP Biomedicals, Cambridge, UK). 1 ml of extraction buffer (sodium dodecyl sulfate 87 mM; sodium phosphate buffer pH 8.0, 200 mM; sodium chloride 100 mM; ethylenediaminetetraacetic acid pH 8.0, 50 mM, Sigma-Aldrich) was added to the sample. The mixture was bead beated at 6 m·s^-1^ for 4 s with a Bead blaster 24 bead beater (Benchmark, Edison, NJ, USA). After centrifugation at 15 000 x *g* for 5 min at 4 ºC, the supernatant was extracted with 850 µl of phenol:chloroform:isoamyl alcohol (25:24:1, Sigma-Aldrich) and then with 800 µl of chloroform:isoamyl alcohol (24:1, Sigma-Aldrich). The nucleic acid extracts were precipitated for 1 h at room temperature with 1 ml of precipitation solution (polyethylene glycol 6000 20%; sodium chloride 2.5 M, Sigma-Aldrich). After centrifugation at 15 000 x *g* for 30 min, pellets were washed with 800 µl of cold 75% ethanol. Pellets containing total nucleic acid extracts were dissolved in 100 µl of nuclease-free water and stored at -80 ºC.

16S rRNA gene amplicon sequencing

The 16S rRNA gene amplicon sequencing analysis of the DNA extracted from the grassland soil samples was performed by MR DNA (Shallowater, TX, USA). Two biological replicates of each condition were analysed. Primer set 515F/806R of the V4 variable region of the 16S rRNA gene (Caporaso *et al.*, 2012) was used in the PCR reaction, with the former being barcoded. The PCR reaction consisted of an initial step of 94 ºC for 3 min, followed by 28 cycles of 94 ºC for 30 s, 53 ºC for 40 s and 72 ºC for 1 min, after which a final elongation step at 72 ºC for 5 min was performed. Samples were later purified using calibrated Ampure XP beads. Purified products were used to prepare an Illumina DNA library. Sequencing was performed on a MiSeq system according to the manufacter’s instructions and data were processed using the MR DNA analysis pipeline, obtaining an average of 47 984 reads per sample with an average length of 300 bp. The data processing included joining the sequences, depleting of the barcodes, removing sequences <150 bp and sequences with ambiguous bases. Resulting sequences were denoised, operational taxonomic units (OTUs) generated and chimeras removed. OTUs were defined by clustering at 3% divergence. Final OTUs were identified taxonomically using BLASTn against a curated database derived from RPDII and NCBI (<http://rdp/cme.msu.edu>, [www.ncbi.nlm.nih.gov](http://www.ncbi.nlm.nih.gov)). Rarefaction curves for all the samples are shown in Supplementary Figure 4.

Metagenomic analysis of the grassland soil samples

DNA extracted from two biological replicates of the grassland soil samples at t=0 and from enrichments with succinate plus MeSH at 7 and 14 days were combined in equal proportions to perform metagenomic analysis. Libraries of DNA extracted from samples were prepared using the Nextera DNA Sample preparation kit (Illumina, San Diego, CA, USA) following the manufacturer's user guide. The initial concentration of DNA was evaluated using the Qubit® dsDNA HS Assay Kit (Life Technologies, Carlsbad, CA, USA). The samples were then diluted to achieve the recommended DNA input of 50 ng at a concentration of 2.5 ng·µl^-1^. Samples then underwent simultaneous fragmentation and addition of adapter sequences. These adapters were incorporated over 5 cycles of PCR. Following the library preparation, the final concentration of the library was measured using the Qubit® dsDNA HS Assay Kit (Life Technologies), and the average library size was determined using the Agilent 2100 Bioanalyzer (Agilent Technologies). The average library size for t=0 samples was 631 bp, for samples enriched with succinate plus MeSH at 7 days, 603 bp, and for samples enriched with succinate plus MeSH at 14 days, 1042 bp. The library was then pooled in equimolar ratios of 2 nM, and 10.5 pM of the library pool was clustered using the cBot (Illumina) and sequenced paired end for 300 cycles using the HiSeq 2500 system (Illumina). Reads were quality-filtered and trimmed using Trimmomatic (Bolger *et al.*, 2014), obtaining an average of 13 909 226 reads per sample with an average length of 151 bp. Metagenomes were then assembled using SPAdes assembler with kmers 55 to 127 (Bankevich *et al.*, 2012), and assemblies were analysed using Quast (Gurevich *et al.,* 2013). N50 values were ~1 kb for all metagenomes assemblies.

The abundance of functional genes in unassembled metagenomes was determined by tBLASTx ([www.ncbi.nlm.nih.gov](http://www.ncbi.nlm.nih.gov)) of selected ratified gene sequences (*mddA*, *ddhA*, *dmoA*, *tmm, megL*) against the raw reads (E≤e^-4^). Each potential MddA, DdhA, DmoA, Tmm, MegL sequence retrieved from the analysis of metagenomes was manually checked by BLASTp against the RefSeq database and discounted as a true sequence of interest if the top hit was not to the ratified sequences described in Supplementary Table 5. Only unique hits were counted. Hit numbers were normalised against read number of the smallest sample, to gene length and to hits of *recA*. Phylogeny of *mddA* unique hits was analysed using Qiime (Caporaso *et al.*, 2010; MacQIIME version 1.9.0) by mapping the reads to a hand-curated reference database of 176 full-length *mddA* sequences, using blat for OTU picking and a cut-off of 45% amino acid identity. Taxonomy of unassembled metagenomes was further analysed using MetaPhlAn (Segata *et al.*, 2012; version 2.2.0).

To determine diversity of *mddA* genes in the assembled metagenomes, contigs were first searched using tBLASTx ([www.ncbi.nlm.nih.gov](http://www.ncbi.nlm.nih.gov)) and selected *mddA* gene sequences (E≤e^-4^). Each potential MddA sequence retrieved from the blast analysis was manually checked by BLASTp as above and only those whose top hit was to the ratified sequences described in Supplementary Table 5 were taken into account. The phylogenetic tree was then reconstructed from *mddA* sequence data using the ARB software package (Ludwig *et al.,* 2004; version 6.0.1). Metagenomics contigs with hits to *mddA* were aligned to a hand-curated reference database of 176 full-length *mddA* sequences. Contig sequences that could not be sufficiently aligned were discarded. *mddA* tree topology was checked by neighbour-joining algorithm using 1 000 bootstrap replicates and Jukes-Cantor correction of distances and was verified with a tree calculated using maximum likelihood.

Statistical analysis

Statistical analyses were performed in R 3.2.3 (R Core Team (2015) using the base stats package, except where otherwise stated. The compositions package (van den Boogaart *et al.,* 2014) was used for appropriate transformation and assessment of the effect of treatments on microbial composition data. Prior to multivariate analyses, data were transformed using an isometric log-ratio (ilr) transformation. A single zero value was found in the Genus-level data, necessitating addition of a small constant (0.01%) to this dataset before transformation. Multivariate microbial response was then assessed by MANOVA (using Pillai’s trace), with soil treatment (7 levels) as the sole explanatory factor. Linear Discriminant Analysis (LDA), using the MASS package (Venables and Ripley, 2002) lda function served as a posthoc test of multivariate treatment differences. Univariate (taxon by taxon) percentage responses to treatments were each subjected to a modified ilr transformation (Flizmoser *et al.,* 2009; equation 5) and analysed by ANOVA, with *p*-values conservatively adjusted (Holm correction) for multiple comparisons. For every significant univariate response thus determined, Tukey HSD tests (95% family-wide confidence levels) were applied to determine posthoc pair-wise differences between treatments.

**Supplementary Figures**

**
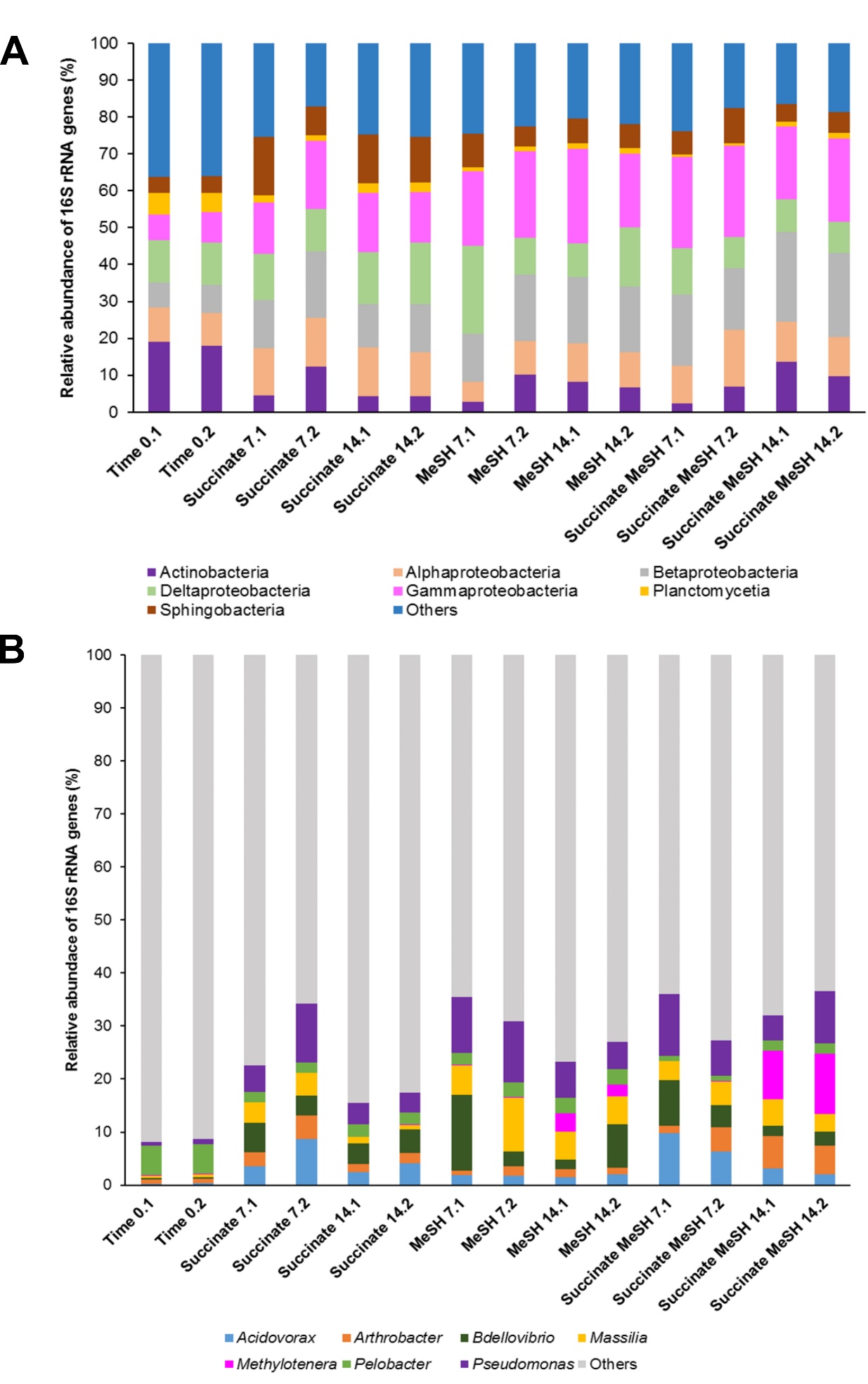
**

**Supplementary Figure 1. Taxonomic profiling of the 16S rRNA gene amplicon sequencing data from grassland soil enrichments.** A: Class level; B: Genus level. Only classes or genera that are ≥5% abundant in at least one of the conditions are represented. Time 0: grassland soil samples at time 0; Succinate 7: enrichments with succinate at 7 days, Succinate 14: enrichments with succinate at 14 days; MeSH 7: enrichments with MeSH at 7 days; MeSH 14: enrichments with MeSH at 14 days; Succinate MeSH 7: enrichments with succinate plus MeSH at 7 days; Succinate MeSH 14: enrichments with succinate plus MeSH at 14 days.

**
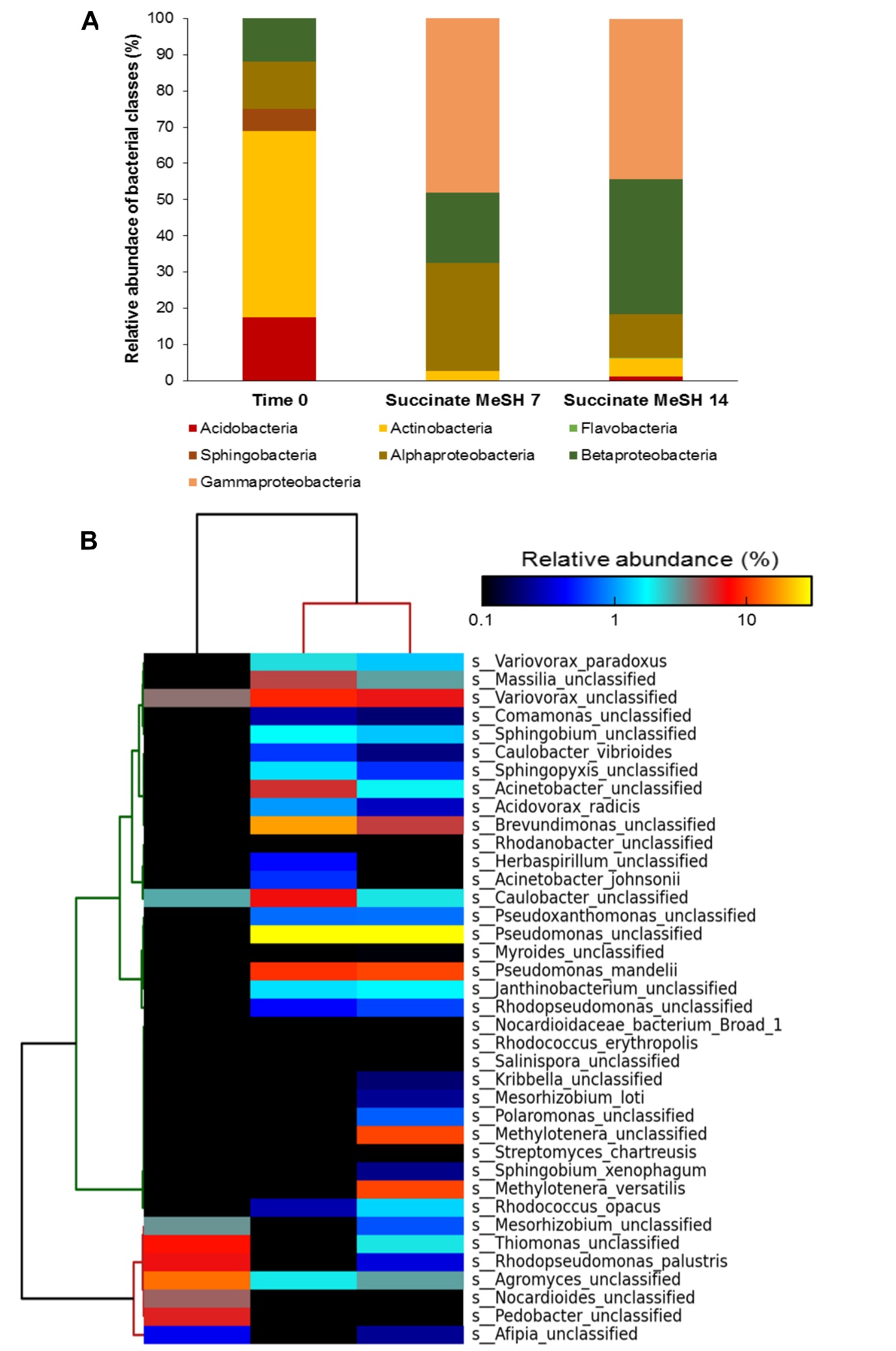
**

**Supplementary Figure 2. Phylogenetic analysis of the metagenomes from the grassland soil by MetaPhlAn.** A: Relative abundance of bacterial classes; B: Abundance for species in logarithmic scale reporting the 50 most abundant clades according to the 90^th^ percentile of the abundance of each clade with a custom colour map. Clustering is performed with average linkage, using Bray-Curtis distance for clades and correlation for samples. Time 0: samples at time 0; Succinate MeSH 7: enrichments with succinate plus MeSH after 7 days; Succinate MeSH 14: enrichments with succinate plus MeSH after 14 days.


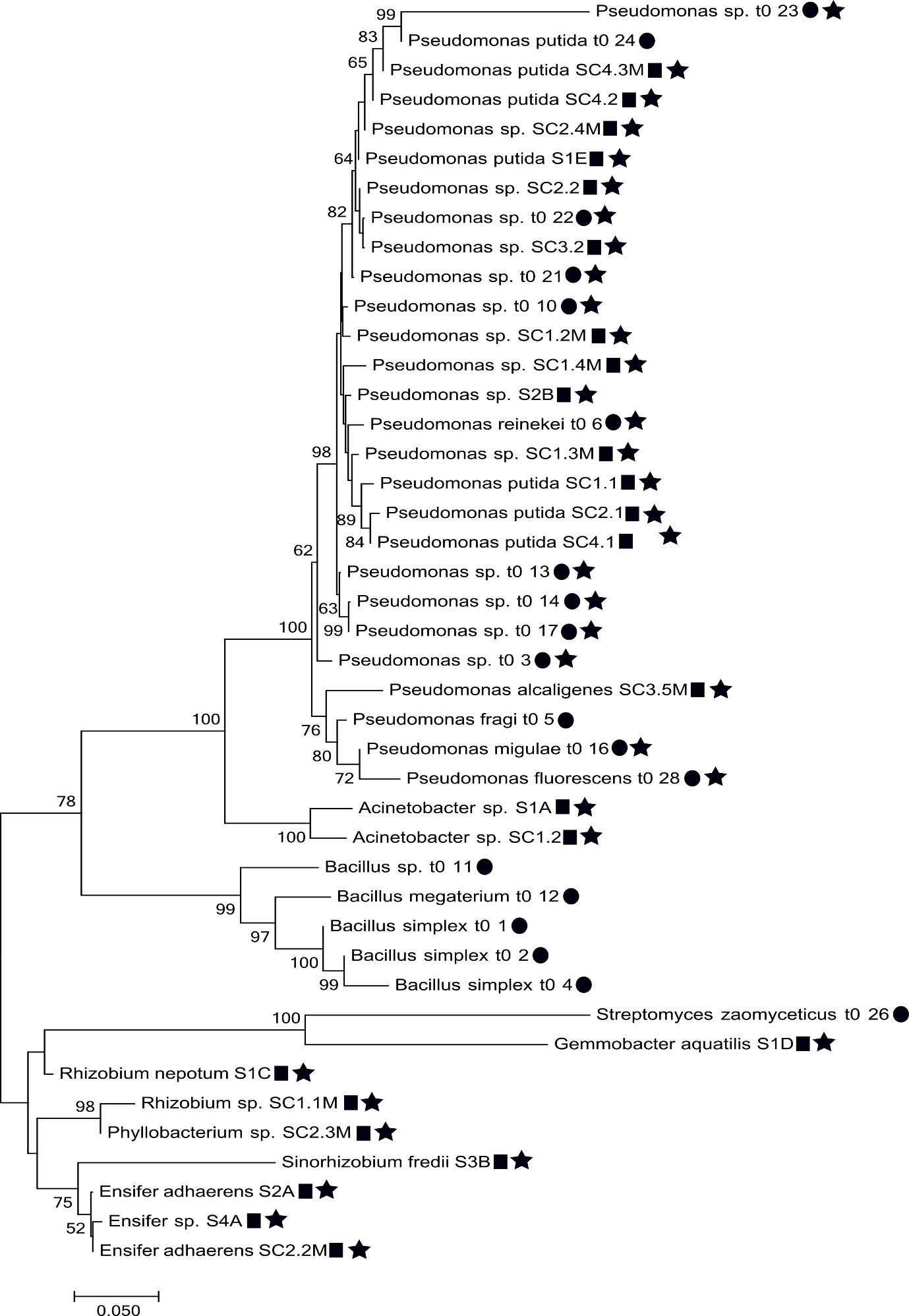


**Supplementary Figure 3. Neighbour-joining phylogenetic tree based on the 16S rRNA gene of the isolates obtained from the grassland soil samples at t=0 and enrichments with succinate plus MeSH at 14 days.** Strains isolated from time 0 are indicated with a circle. Strains isolated after 14 days of enrichment with succinate and MeSH are indicated with a square. Isolates that are Mdd^+^ are indicated with a star. Bar, 0.05 substitutions per nucleotide position. Bootstrap values ≥50% (based on 1 000 replicates) are shown at branch points.


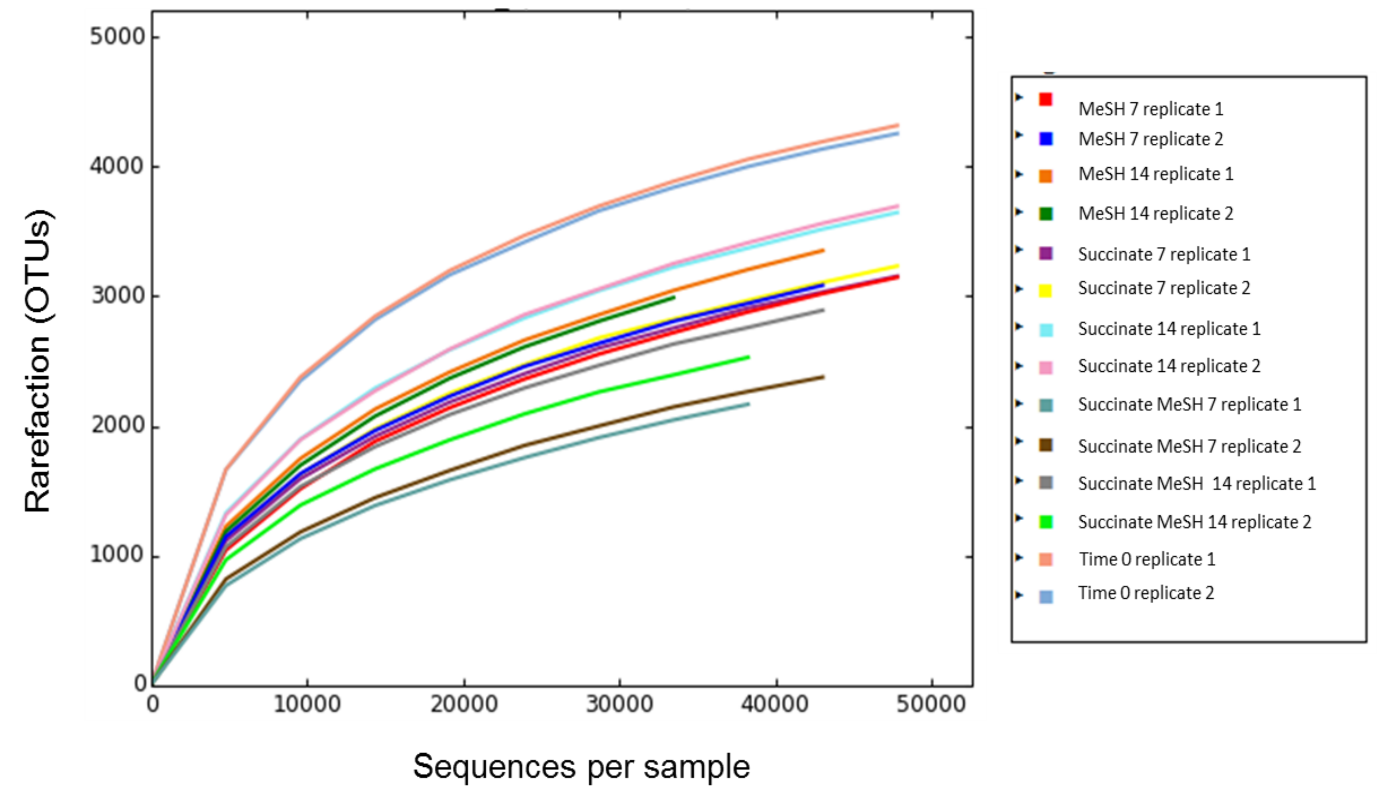


**Supplementary Figure 4. Rarefaction curves of the 16S rRNA gene amplicon sequencing analysis of the grassland soil samples.** Total OTUs were generated by 3% divergency. Total sample richness estimates were calculated by the observed OTUs. Time 0: samples at time 0; MeSH 7: enrichment with MeSH at 7 days; MeSH 14: enrichment with MeSH at 14 days; Succinate 7: enrichment with succinate at 7 days; Succinate 14: enrichment with succinate at 14 days; Succinate MeSH 7: enrichment with succinate plus MeSH at 7 days; Succinate MeSH 14: enrichment with succinate plus MeSH at 14 days.

**Supplementary Tables**

**Supplementary Table 1. Characteristics of the environments tested for DMS production from MeSH.**

| **Environment** | **Location** | **Coordinates** | **pH** |
| --- | --- | --- | --- |
| Grassland soil A | Norwich | 52º37’09.8”N, 1º14’20.4”E | 6.7 |
| Grassland soil B | Norwich | 52º37’10.5”N, 1º14’02.2”E | 6.5 |
| Forest soil | Norwich | 52º37’22.6”N, 1º13’53.1”E | 6.9 |
| Maize field | Scratby | 52º40’48.5”N, 1º42’19.9”E | 6.2 |
| Barley field | Mulbarton | 52º55’62.7”N, 1º24’39.3”E | 5.9 |
| Lake sediment | University of East Anglia Broad | 52º37’0.7.7”N, 1º14’17.0”E | 6.3 |
| River sediment | River Yare | 52º37’46.6”N, 1º14’00.5”E | 6.3 |
| Beach sand A | Caister-on-Sea | 52º39’07.5”N, 1º44’00.9”E | 7.5 |
| Beach sand B | Winterton-on-Sea | 52°43'03.1"N, 1°42'01.6"E | 7.8 |
| Seawater A | Caister-on-Sea | 52°39'07.5"N, 1°44'00.9"E | 8.3 |
| Seawater B | Winterton-on-Sea | 52°43'03.2"N, 1°42'02.2"E | 7.9 |
| Marine sediment A | Stiffkey | 52º57’54.0’’N, 0º55’31.0’’E | 7.8 |
| Marine sediment B | Great Yarmouth | 52°36'52.4"N, 1°42'56.5"E | 8.0 |

**Supplementary Table 2. MeSH and DMS consumption by *Methylotenera mobilis* JLW8^T^.** The concentrations of MeSH and DMS in the headspace in medium-only controls and *M. mobilis* cultures were measured at time 0 (t=0) and after 24 h (t=24) of incubation at 30 ºC. Results shown are the average of three biological replicates with their respective standard deviations.

|  | nmol MeSH | | nmol DMS | |
| --- | --- | --- | --- | --- |
|  | t=0 | t=24 | t=0 | t=24 |
| Medium control | 458.2 ± 13.4 | 388.8 ± 34.8 | 1.2 ± 0.05 | 1.3 ± 0.002 |
| *M. mobilis* JLW8^T^ | 581.7 ± 26.0 | 38.1 ± 0.9 | 1.1 ± 0.02 | 0.04 ± 0.005 |

**Supplementary Table 3. MeSH and DMS production by the isolates obtained from the grassland soil.** Table shows MeSH and DMS produced by each isolate in Y minimal medium alone, Y medium supplemented with Met (0.5 mM) or Y medium supplemented with MeSH (0.3 µmol). Results shown are the average of three biological replicates with their respective standard deviations. Isolates from time 0 are indicated with (*). Strains obtained from the succinate plus MeSH enrichment after 14 days of incubation are indicated with (#). ND, Not detected. Rates of MeSH and DMS production are expressed as nmol·min^-1^·mg prot^-1^. The percentage of MeSH converted to DMS by each isolate is indicated in brackets.

| **Isolate** | **Y medium** | | | **Y medium+Met** | | | **Y medium+MeSH** |
| --- | --- | --- | --- | --- | --- | --- | --- |
|  | **MeSH** | **DMS** | | **MeSH** | **DMS** | | **DMS** |
| *Bacillus simplex* t0_1* | ND | | ND | 30.31 ± 6.69 | | ND | ND |
| *Bacillus simplex* t0_2* | ND | | ND | 17.73 ± 4.08 | | ND | ND |
| *Pseudomonas* sp. t0_3* | ND | | 0.02 ± <0.01 | 2.68 ± 0.71 | | 0.08 ± 0.02 | 0.05 ± 0.02 (0.91%) |
| *Bacillus simplex* t0_4* | ND | | ND | 4.87 ± 0.12 | | ND | ND |
| *Pseudomonas fragi* t0_5* | ND | | ND | 22.57 ± 0.53 | | ND | ND |
| *Pseudomonas reinekei* t0_6* | ND | | ND | 0.36 ± 0.04 | | 0.07 ± 0.01 | 0.10 ± <0.01 (1.08%) |
| *Pseudomonas* sp. t0_10* | ND | | ND | 0.53 ± 0.01 | | 0.08 ± 0.01 | 0.14 ± 0.05 (0.90%) |
| *Bacillus* sp. t0_11* | ND | | ND | ND | | ND | ND |
| *Bacillus megaterium* t0_12* | ND | | ND | 4.90 ± 0.32 | | ND | ND |
| *Pseudomonas* sp. t0_13* | ND | | ND | 1.50 ± 0.07 | | 0.06 ± <0.01 | 0.35 ± 0.05 (0.61%) |
| *Pseudomonas* sp. t0_14* | ND | | ND | 1.00 ± 0.17 | | 0.06 ± 0.03 | 0.15 ± 0.03 (0.47%) |
| *Pseudomonas migulae* t0_16* | ND | | ND | 1.42 ± 0.01 | | 0.06 ± <0.01 | 0.14 ± 0.01 (0.47%) |
| *Pseudomonas* sp. t0_17* | ND | | ND | 1.51 ± 0.19 | | 0.08 ± 0.01 | 0.15 ± 0.01 (0.47%) |
| *Pseudomonas* sp. t0_21* | ND | | ND | ND | | 0.13 ± 0.02 | 0.22 ± 0.03 (0.51%) |
| *Pseudomonas* sp. t0_22* | ND | | ND | ND | | 0.05 ± 0.01 | 0.11 ± 0.01 (0.75%) |
| *Pseudomonas* sp. t0_23* | ND | | ND | ND | | 0.03 ± <0.01 | 0.07 ± 0.01 (0.43%) |
| *Pseudomonas putida* t0_24* | ND | | ND | 0.30 ± 0.02 | | ND | ND |
| *Streptomyces zaomyceticus* t0_26* | ND | | ND | 2.76 ± 0.16 | | ND | ND |
| *Pseudomonas fluorescens* t0_28* | ND | | ND | ND | | 0.07 ± 0.01 | 0.30 ± 0.09 (0.97%) |
| *Acinetobacter* sp. S1A # | ND | | ND | 1.23 ± 0.70 | | 0.02 ± <0.01 | 0.10 ± 0.01 (0.11%) |
| *Rhizobium* *nepotum* S1C # | ND | | ND | 6.52± 1.07 | | <0.01 | <0.01 (0.04 %) |
| *Gemmobacter aquatilis* S1D # | ND | | ND | 4.67 ± 0.42 | | 0.01 ± <0.01 | 0.04 ± 0.01 (0.08%) |
| *Pseudomonas putida* S1E # | ND | | ND | 0.42 ± 0.15 | | 0.01 ± <0.01 | 0.02 ± <0.01 (0.06%) |
| *Ensifer adhaerens* S2A # | ND | | ND | 3.53 ± 0.58 | | 0.02 ± <0.01 | 0.04 ± <0.01 (0.12%) |
| *Pseudomonas* sp. S2B # | ND | | ND | 0.51 ± <0.01 | | 0.01 ± <0.01 | 0.03 ± 0.01 (0.08%) |
| *Sinorhizobium fredii* S3B # | ND | | ND | 17. 94 ± 2.56 | | 0.07 ± 0.01 | 0.03 ± <0.01 (0.07%) |
| *Ensifer* sp. S4A # | ND | | ND | 28.08 ± 1.76 | | 0.04 ± 0.01 | 0.03 ± <0.01 (0.07%) |
| *Pseudomonas* *putida* SC1.1 # | 0.57 ± 0.06 | | 0.01 ± <0.01 | 27.50 ± 1.19 | | 0.01 ± <0.01 | 0.04 ± 0.01 (0.12%) |
| *Acinetobacter* sp. SC1.2 # | ND | | ND | ND | | ND | 0.10 ± 0.02 (0.20%) |
| *Pseudomonas putida* SC2.1 # | 0.55 ± 0.01 | | 0.01 ± <0.01 | 28.80 ± 4.10 | | 0.02 ± <0.01 | 0.02 ± <0.01 (0.09%) |
| *Pseudomonas* sp. SC2.2 # | ND | | 0.02 ± <0.01 | ND | | 0.03 ± <0.01 | 0.02 ± <0.01 (0.17%) |
| *Pseudomonas* sp. SC3.2 # | ND | | 0.02 ± <0.01 | 0.76 ± 0.01 | | 0.02 ± 0.01 | 0.05 ± 0.01 (0.20%) |
| *Pseudomonas* *putida* SC4.1 # | 0.62 ± 0.12 | | 0.02 ± 0.01 | 4.77 ± 0.46 | | 0.02 ± <0.01 | 0.03 ± <0.01 (0.11%) |
| *Pseudomonas* *putida* SC4.2 # | ND | | 0.03 ± <0.01 | 0.64 ± 0.91 | | 0.03 ± 0.01 | 0.04 ± <0.01 (0.18%) |
| *Rhizobium* sp. SC1.1M # | ND | | ND | 12.93 ± 0.72 | | 0.02 ± 0.01 | 0.03 ± <0.01 (0.55%) |
| *Pseudomonas* sp. SC1.2M # | ND | | ND | 8.64 ± 1.22 | | 0.05 ± 0.01 | 0.06 ± 0.02 (0.57%) |
| *Pseudomonas* sp. SC1.3M # | ND | | 0.02 ± <0.01 | 0.75 ± 0.28 | | 0.06 ± <0.01 | 0.06 ± 0.01 (0.71%) |
| *Pseudomonas* sp. SC1.4M # | ND | | 0.01 <0.01 | 6.33 ± 1.27 | | 0.07 ± 0.01 | 0.04 ± 0.01 (0.51%) |
| *Ensifer adhaerens* SC2.2M # | ND | | ND | 3.29 ± 0.14 | | 0.05 ± <0.01 | 0.05 ± 0.01 (0.35%) |
| *Phyllobacterium* sp. SC2.3M # | ND | | ND | 0.39 ± 0.15 | | 0.01 ± <0.01 | 0.21 ± 0.04 (2.38 %) |
| *Pseudomonas* sp. SC2.4M # | ND | | 0.01 ± <0.01 | 1.96 ± 0.66 | | 0.15 ± 0.09 | 0.10 ± 0.01 (1.74 %) |
| *Pseudomonas* *alcaligenes* SC3.5M # | ND | | ND | 3.46 ± 0.89 | | 0.08 ± 0.02 | 0.11 ± 0.04 (1.58%) |
| *Pseudomonas putida* SC4.3M # | ND | | 0.01 ± <0.01 | 1.83 ± 0.22 | | 0.11 ± 0.05 | 0.04 ± <0.01 (1.27%) |

**Supplementary Table 4. Comparison of normalized values of *mddA*, *megL*, *ddhA*, *dmoA* and *tmm* sequences in grassland soil unassembled metagenomes.** Unique hits of the target genes were normalised to the read number of the smallest sample, to gene length and to unique hits of *recA* to predict the percentage of bacteria that contain these genes. Time 0: samples at time 0; Succinate MeSH 7: enrichments with succinate plus MeSH after 7 days; Succinate MeSH 14: enrichments with succinate plus MeSH after 14 days.

| Gene | Time 0  (% of bacteria) | Succinate MeSH 7  (% of bacteria) | Succinate MeSH 14  (% of bacteria) |
| --- | --- | --- | --- |
| *mddA* | 35.9 | 19.5 | 25.3 |
| *ddhA* | 6.0 | 3.9 | 4.1 |
| *dmoA* | 10.0 | 7.5 | 3.2 |
| *tmm* | 2.2 | 1.2 | 1.8 |
| *megL* | 78.0 | 54.6 | 50.4 |

**Supplementary Table 5. Selected ratified proteins used to confirm sequences obtained from the metagenomics analysis as functional genes of interest.**

| Refseq | Accession number | Microorganism | Reference |
| --- | --- | --- | --- |
| MddA | AJE75769.1  WP_008148420.1  NP_772381.1  NP_767858.1  YP_001803274.1  NP_217755.1 | *Pseudomonas deceptionensis*  *Pseudomonas* sp. GM41(2012)  *Bradyrhizobium diazzoefficiens* USDA 110  *Bradyrhizobium diazzoefficiens* USDA 110  *Cyanothece* sp. ATCC 51142  *Mycobacterium tuberculosis* H37Rv | Carrión *et al.,* 2015 |
| DmoA | E9JFX9.1 | *Hyphomicrobium sulfonivorans* | Boden *et al.*, 2011 |
| DddhA | Q8GPG4.1  Q8GPG3.1 | *Rhodovulum sulfidophilum* | McDevitt *et al.,* 2002 |
| Tmm | ACK52489.1  AAV94838.1  EAQ26624.1 | *Methylocella silvestris* BL2  *Ruegeria pomeroyi* DSS-3  *Roseovarius* sp. 217 | Chen *et al.,* 2011  Lidbury *et al.,* 2016  Lidbury *et al.,* 2016 |
| MegL | P13254.2  KMM80926.1  Q8L0X4.1  AAO46884.1  AAV54600.1 | *Pseudomonas putida*  *Pseudomonas deceptionensis*  *Fusobacterrium nucleatum*  *Citrobacter freundii*  *Brevibacterium linens* | Inoue *et al.,* 1995  Carrión *et al*., 2015  Yoshimura *et al.,* 2002  Manukhov *et al.*, 2005  Amarita *et al.,* 2004 |

**References**

Amarita F, Yvon M, Nardi M, Chambellon E, Delettre J, Bonnarme P. Identification and functional analysis of the gene encoding methionine-gamma-lyase in *Brevibacterium linens. Appl Environ Microbiol* **70:** 7348-7354.

Bankevich A, Nurk S, Antipov D, Gurevich AA, Dvorkin M, Kulikov, AS *et al.* (2012). SPAdes: A new genome assembly algorithm and its applications to single-cell sequencing. *J Comput Biol* **19:** 455-477.

Bolger AM, Lohse M, Usadel B. (2014). Trimmomatic: a flexible trimmer for Illumina sequence data. *Bioinformatics* **30:** 2114-2220.

Caporaso JG, Lauber CL, Walters WA, Berg-Lyons D, Huntley J, Fierer N *et al*. (2012). Ultra-high-throughput microbial community analysis on the Illumina HiSeq and MiSeq platforms. *ISME J* **6:** 1621-1624.

Caporaso JG, Kuczynski J, Stombaugh J, Bittinger K, Bushman FD, Costello EK *et al.* (2010). QIIME allows analysis of high-throughput community sequencing data. *Nat Methods* **7:** 335-336.

Chen Y, Patel NA, Crombie A, Scrivens JH, Murrell JC. (2011). Bacterial flavin-containing monooxygenase is trimethylamine monooxygenase. *PNAS* **108:** 17791-17796.

Delaney NF, Kaczmarek ME, Ward LM, Swanson PK, Lee M, Marx CJ. (2013) Development of an optimized medium, strain and high-throughput culturing methods for *Methylobacterium extorquens*. *Plos One* **8:**e62957.

DeLong EF. (1992). Archaea in coastal marine environments. *Proc Natl Acad Sci USA* **89:** 5685-5689.

Gurevich A, Saveliev V, Vyahhi N, Tesler G. (2013). QUAST: quality assessment tool for genome assemblies. *Bioinformatics* **29:** 1072-1075.

Inoue H, Inagaki K, Sugimoto M, Esaki N, Soda K, Tanaka H. (1995). Structural analyisis of the L-methionine gamma-lyase gene from *Pseudomonas putida. J Biochem* **117:** 1120-1125.

Kalyuzhnaya MG, Bowerman S, Lara JC, Lidstrom ME, Chistoserdova L. (2006). *Methylotenera mobilis* gen. nov., sp. nov., an obligately methylamine-utilizing bacterium within the family *Methylophilaceae*. *Int J Syst Evol Microbiol* **56:** 2819–2823.

Lane DJ, Pace B, Olsen GJ, Stahl DA, Sogin ML, Pace NR. (1985). Rapid determination of 16S ribosomal RNA sequences for phylogenetic analyses. *Proc Natl Acad Sci USA* **82:** 6955–6959.

Ludiwg W, Strunk O, Westram R, Richter L, Meier H, Yadhukumar *et al.* (2004). ARB: a software environment for sequence data. *Nucleic Acids Res* **32:** 1363-1371.

Manukhov IV, Mamaeva DV, Rastorguev SM, Faleev NG, Morozova EA, Demidkina TV *et al*. (2005). A gene encoding L-methionine γ-lyase is present in *Enterobacteriaceae* family genomes: identification and characterization of *Citrobacter freundii* L-methionine γ-lyase. *J Bacteriol* **187:** 3889-3893.

R Development Core Team (2011), R: A Language and Environment for Statistical Computing. Vienna, Austria : the R Foundation for Statistical Computing. ISBN: 3-900051-07-0.

Segata N, Waldron L, Ballarini A, Narasimhan V, Jousson O, Huttenhower C. (2012) Metagenomic microbial community profiling using clade-specific marker genes. *Nat Methods* **9:** 911-814.

Van den Boogaart KG, Tolosana-Delgado R. (2008). “Compositions”: a unified R package to analyze compositional data. *Comput Geosci* **34:** 320-338.

Venables WN, Ripley BD. (2002) Modern Applied Statistics with S. Fourth Edition. Springer, New York. ISBN 0-387-95457-0.

Yoshimura M, Nakano Y, Fukamachi H, Koga T. (2002). 3-Chloro-DL-alanine resistance by L-methionine-alpha-deamino-gamma-mercaptomethane-lyase activity. *FEBS Lett* **523:** 119-122.
